# Acute Gastric Dilatation in Duchenne Muscular Dystrophy: A Case Report

**DOI:** 10.7759/cureus.81440

**Published:** 2025-03-29

**Authors:** Yhonatan R Ramírez-Guerra, Marco A Arizmendi-Villarreal, Luis F Zorrilla-Núñez, Gerardo E Muñoz-Maldonado

**Affiliations:** 1 Department of General Surgery, Hospital Universitario Dr. José Eleuterio González, Monterrey, MEX

**Keywords:** abdominal pain, acute gastric dilatation, duchenne muscular dystrophy, gastroparesis, prokinetic

## Abstract

Duchenne muscular dystrophy (DMD) is an X-linked hereditary disease characterized by a structural defect in dystrophin, affecting muscle cells, leading to their progressive degeneration. Gastrointestinal manifestations are uncommon but can contribute to significant morbidity and mortality. Acute gastric dilatation is a rare clinical entity and the most severe complication of this spectrum. We present the case of a 16-year-old male patient with DMD who developed acute gastric dilatation, managed with decompression via nasogastric tube, intravenous fluids, and prokinetics, resulting in clinical improvement.

## Introduction

Duchenne muscular dystrophy (DMD) is an X-linked hereditary disease with a global prevalence of 4.8 per 100,000 people. It is characterized by a structural defect in dystrophin, a muscle protein that connects F-actin in the cytoskeleton to the extracellular matrix. Together with other associated proteins, dystrophin helps stabilize muscle fibers during contraction and relaxation. Gastrointestinal manifestations in the context of DMD are relatively uncommon when compared to cardiovascular or respiratory complications, which are the two leading causes of death in this disease [1,2]. Deficiency of functional dystrophin in the smooth muscle of the stomach and small bowel leads to gastrointestinal symptoms, ranging from chronic constipation and/or dyspepsia to acute refractory gastroparesis, which can result in high mortality if not detected and treated in time [3,4].

Acute gastric dilatation is a consequence of refractory gastroparesis due to the absence of dystrophin in the smooth muscle of the digestive tract, manifested as delayed gastric emptying, increased diameter of the stomach and small bowel, and the absence of evacuations. It is a clinical entity that can compromise life and responds adequately to prokinetics, gastric decompression via nasogastric tube, and fluid resuscitation [1,3,4].

As it manifests as a rare complication, there are few case reports on acute gastric dilatation in DMD patients, and its management is not well clarified, with a preference for medical treatment with prokinetics. We present the case of a 16-year-old patient with a history of DMD who presented with acute gastric dilatation and resolved with medical treatment.

## Case presentation

A 16-year-old male patient with a clinical history of DMD, diagnosed at five years of age with genetic testing showing a de novo mutation, presents to the emergency department with generalized abdominal pain, vomiting, and absence of evacuations for the past 72 hours. Upon admission, the patient was afebrile, tachycardic with a heart rate of 112 bpm, and had a blood pressure of 110/60 mmHg. Physical examination revealed malnutrition with a body mass index of 18.6 kg/m², in a forced flexion position, cachexia, and hypotrophic extremities. The abdomen was slightly distended, soft, and tender to palpation with decreased peristalsis. Rectal examination showed decreased anal sphincter tone and no fecal matter in the rectal ampulla. Laboratory findings are summarized in Table 1.

**Table 1:** Laboratory and venous blood gas results at admission PaO₂: partial pressure of oxygen in venous blood; PaCO₂: partial pressure of carbon dioxide in venous blood; HCO₃⁻: bicarbonate

Parameter	Value	Reference range
Hemoglobin (g/dL)	15.3	12.20-18.10
White blood cells (10^3^/µL)	19.0	4.0-11.0
Platelets (10^3^/µL)	593.0	142.0-424.0
Prothrombin time (seconds)	15.5	10.43-12.80
Activated partial thromboplastin time (seconds)	27.1	25.1-36.0
International normalized ratio (INR)	1.40	
Creatinine (mg/dL)	0.2	0.6-1.4
Blood urea nitrogen (mg/dL)	23	6-20
Albumin (g/dL)	3.6	3.5-5.0
Sodium (mEq/L)	148	135-145
Chloride (mEq/L)	93.0	98-107
Potassium (mEq/L)	2.7	3.5-5.0
pH	7.48	7.35-7.45
PaO₂ (mmHg)	43	80-100
PaCO₂ (mmHg)	37	35-45
HCO₃⁻ (mmol/L)	27.6	21-28
Lactate (mmol/L)	1.3	0.5-2.2

**Figure 1:**
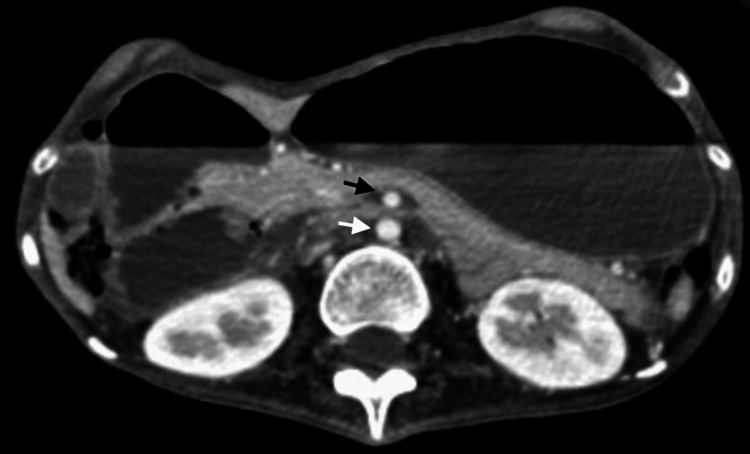
Abdominopelvic CT scan (axial view) revealing a distended stomach and the first part of the duodenum, with a narrowed aortomesenteric distance (8 mm), which is indicative of Wilkie syndrome CT: computed tomography The white arrow shows the abdominal aorta; the black arrow shows the superior mesenteric artery

**Figure 2:**
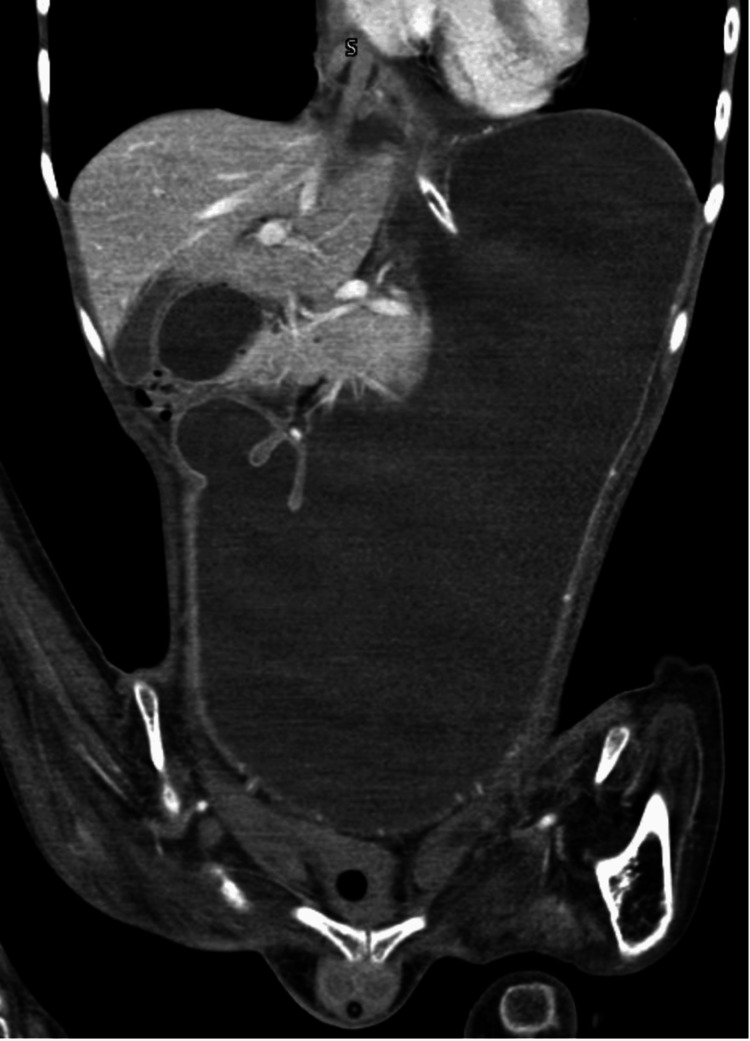
Abdominopelvic CT scan (coronal view) showing a dilated stomach and duodenum, with extension into the pelvic cavity CT: computed tomography

Abdominopelvic computed tomography (CT) scan showed dilation of the gastric chamber extending to the pelvic cavity and enlargement of the first portion of the duodenum proximal to the superior mesenteric artery (Figures 1, 2).

Intravenous fluid therapy was initiated along with electrolyte replacement. A nasogastric tube was inserted, draining 2000 mL of gastric contents, showing a decrease in the abdominal perimeter. Subsequently, intravenous fluids were substituted with central parenteral nutrition. Endoscopy revealed isolated erosions in the gastric antrum and duodenal bulb up to the second portion of the duodenum. After 24 hours, enteral nutrition via nasojejunal tube was well tolerated, and cisapride was administered at a dose of 10 mg every eight hours; peristaltic movements were observed 12 hours after the first dose of prokinetic.

**Figure 3:**
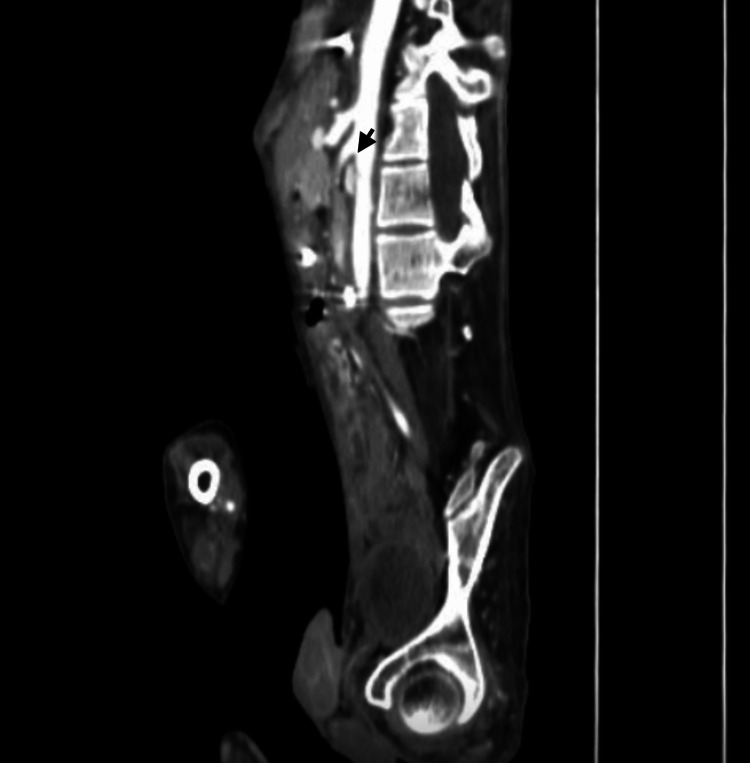
Abdominopelvic CT scan (sagittal view) showing reduced dilation of the stomach and duodenum, while the narrow aortomesenteric distance remains unchanged (black arrow) CT: computed tomography

A progressive oral diet was started, preserving normal bowel movements and stool deposition. A control CT scan showed a reduction in gastric and duodenal dilation without change in the aortomesenteric distance, ruling out the suspicion of superior mesenteric artery syndrome (Wilkie syndrome) (Figure 3).

After 10 days of hospitalization, the enteral tubes were removed, and the patient was discharged with outpatient follow-up without recurrence of the condition.

## Discussion

DMD is a rare condition that impacts multiple bodily systems. Mutations in the DMD gene (responsible for encoding dystrophin) disrupt the protein's reading frame, generating a premature stop codon that weakens the sarcolemma in response to muscular contraction forces. This leads to necrosis and replacement of muscle tissue with connective and adipose tissue [5,6]. Clinically, it manifests after a relatively normal development period. At around three to five years of age, motor deficits begin, and patients typically become wheelchair-dependent by age 12. Mortality is primarily due to cardiovascular issues and secondarily from ventilatory problems. Our patient began experiencing motor issues, such as difficulty walking, at age four. Gastrointestinal manifestations in the context of DMD are associated with smooth muscle atrophy due to the absence of dystrophin. The spectrum can vary, and these patients are more prone to conditions like gastroesophageal reflux and esophagitis, due to the involvement of the pharyngeal and hypopharyngeal muscles. Constipation results from hypomotility of the intestinal smooth muscle, which may be exacerbated by the lack of mobility in these patients [3,4].

A case-control study involving 55 pediatric patients concluded that upper gastrointestinal tract disorders, such as oropharyngeal dysfunction, esophageal issues, and gastric problems, are more common in DMD patients than in healthy individuals [2]. In our case, the patient has been wheelchair-dependent since age 11 and reports having bowel movements every two days, as mentioned by his family.

Histopathological findings of the gastrointestinal tract in patients with DMD showed significant gastric abnormalities, including edema and muscular atrophy with depletion of muscle cells and replacement by connective and fibrous tissue, like the process affecting skeletal muscle in these same patients, without evidence of involvement of the enteric nervous plexus [7,8].

Acute gastric dilatation is a rare manifestation that results in distention of the gastric chamber and small intestine, characterized by a pseudo-obstruction that can be associated with metabolic acidosis and high mortality if not detected promptly. In this case, our patient was admitted to the emergency department, and metabolic acidosis was ruled out, as shown in Table 1. However, prompt electrolyte replenishment was required to correct the hypokalemia and prevent a potentially fatal outcome.

Crowe described the first case of this complication in 1961 in a nine-year-old patient with progressive muscular dystrophy, treated conservatively with nasogastric tube insertion and intravenous fluids [9]. The first reports in the context of DMD were by Robin and Falewski in 1963, describing recurrent episodes of acute gastric dilatation in pediatric patients [10]. Other reports of this entity described medical management with gastric decompression and electrolyte repletion, with recurrence after two weeks [11]. In 1996, Bensen et al. reported a 19-year-old patient with DMD and acute gastric dilatation managed with H2 antagonists and cisapride, showing favorable results and no recurrence of the condition. On the other hand, Chung et al. suggested that the use of prokinetic medications might help improve gastrointestinal symptoms and gastroparesis in DMD patients [12,13]. In 2000, Lunshof et al. described the case of a 15-year-old patient with acute gastric dilatation that resolved adequately without prokinetic medications and without recurrence [14]. Managing these complications can be challenging for healthcare providers, especially without prior knowledge and a strong clinical suspicion.

Metoclopramide is a dopamine receptor 2 (D2) antagonist, and it is the first-line agent in the pharmacological treatment of gastroparesis [15]. Cisapride is a prokinetic drug that is administered orally and acts as an agonist of serotonin 5HT4 receptors, as well as an antagonist of the 5HT3 receptor. In our case, cisapride was preferred over metoclopramide for the treatment of gastroparesis secondary to DMD due to its safety profile, with a lower incidence of adverse effects on the central nervous system and extrapyramidal symptoms [16]. Although there are no guidelines on the treatment of gastrointestinal manifestations in DMD, medical treatment is most frequent. It includes adequate water intake with a high-fiber diet and the use of stool softeners. The use of proton pump inhibitors for gastroesophageal reflux is well described in the literature, and it has been shown to be effective and safe in these patients [3]. In front of a patient with acute gastric dilatation, prokinetics and nasogastric tube decompression with electrolyte repletion might be indicated [3,4,9-14].

## Conclusions

The gastrointestinal issues associated with DMD encompass a broad range of conditions, which can vary in severity and significantly affect patients’ quality of life. Acute gastric dilatation, a rare but potentially fatal complication resulting from gastroparesis due to DMD, has been reported in a few cases since 1961 and leads to electrolyte imbalances and metabolic acidosis. There are no established guidelines or prospective studies to recommend the best treatment approach for these cases, but gastric decompression and the use of prokinetic drugs may offer a suitable solution for this life-threatening condition. Currently, there is no evidence to suggest that surgery would be beneficial in treating acute gastric dilatation or gastroparesis in these patients, though it may be an option in refractory cases.
